# Calcium mishandling in absence of primary mitochondrial dysfunction drives cellular pathology in Wolfram Syndrome

**DOI:** 10.1038/s41598-020-61735-3

**Published:** 2020-03-16

**Authors:** Chiara La Morgia, Alessandra Maresca, Giulia Amore, Laura Ludovica Gramegna, Michele Carbonelli, Emanuela Scimonelli, Alberto Danese, Simone Patergnani, Leonardo Caporali, Francesca Tagliavini, Valentina Del Dotto, Mariantonietta Capristo, Federico Sadun, Piero Barboni, Giacomo Savini, Stefania Evangelisti, Claudio Bianchini, Maria Lucia Valentino, Rocco Liguori, Caterina Tonon, Carlotta Giorgi, Paolo Pinton, Raffaele Lodi, Valerio Carelli

**Affiliations:** 1 IRCCS Istituto delle Scienze Neurologiche di Bologna, UOC Clinica Neurologica, Bologna, Italy; 2 Dipartimento di Scienze Biomediche e Neuromotorie, Università di Bologna, Bologna, Italy; 3 IRCCS Istituto delle Scienze Neurologiche di Bologna, UO Diagnostica Funzionale Neuroradiologica, Bologna, Italy; 4 Department of Morphology, Surgery and Experimental Medicine, Section of Pathology, Oncology and Experimental Biology, Laboratory for Technologies of Advanced Therapies (LTTA), University of Ferrara, Ferrara, Italy; 5 Maria Cecilia Hospital, GVM Care & Research, 48033 Cotignola Ravenna, Italy; 6 Ospedale Oftalmico Roma, Rome, Italy; 7 Studio Oculistico D’Azeglio, Bologna, Italy; 8 IRCCS G.B. Bietti Foundation, Rome, Italy

**Keywords:** Neurodegenerative diseases, Neurodegeneration

## Abstract

Wolfram syndrome (WS) is a recessive multisystem disorder defined by the association of diabetes mellitus and optic atrophy, reminiscent of mitochondrial diseases. The role played by mitochondria remains elusive, with contradictory results on the occurrence of mitochondrial dysfunction. We evaluated 13 recessive WS patients by deep clinical phenotyping, including optical coherence tomography (OCT), serum lactic acid at rest and after standardized exercise, brain Magnetic Resonance Imaging, and brain and muscle Magnetic Resonance Spectroscopy (MRS). Finally, we investigated mitochondrial bioenergetics, network morphology, and calcium handling in patient-derived fibroblasts. Our results do not support a primary mitochondrial dysfunction in WS patients, as suggested by MRS studies, OCT pattern of retinal nerve fiber layer loss, and, in fibroblasts, by mitochondrial bioenergetics and network morphology results. However, we clearly found calcium mishandling between endoplasmic reticulum (ER) and mitochondria, which, under specific metabolic conditions of increased energy requirements and in selected tissue or cell types, may turn into a secondary mitochondrial dysfunction. Critically, we showed that Wolframin (WFS1) protein is enriched at mitochondrial-associated ER membranes and that in patient-derived fibroblasts WFS1 protein is completely absent. These findings support a loss-of-function pathogenic mechanism for missense mutations in *WFS1*, ultimately leading to defective calcium influx within mitochondria.

## Introduction

Wolfram Syndrome (WS) is a rare genetic disorder also known as DIDMOAD, i.e. Diabetes Insipidus (DI), Diabetes Mellitus (DM), Optic Atrophy (OA) and Deafness^1^. Additional neurological features include brainstem atrophy, cerebellar ataxia, peripheral neuropathy, cognitive deterioration and epilepsy^2^. The onset of OA and DM, the minimal diagnostic criteria for WS, is typically within the second decade.

The large majority of WS patients carry recessive mutations in the *WFS1* gene encoding for the Wolframin (WFS1) protein^3^. WFS1 is associated with the endoplasmic reticulum (ER)^4^ and cellular and animal model studies involved Wolframin in the ER stress response, regulation of calcium homeostasis and Na/K ATPase function^5^. Due to the clinical features resembling a mitochondrial disorder^6^ and reports of mitochondrial DNA (mtDNA) abnormalities in WS^7,8^, a possible mitochondrial dysfunction has been debated with a long-standing controversy^9–12^. In 1998, the identification of Wolfram Syndrome 1 (*WFS1)* gene mutations apparently resolved this issue^3^. However, recent reports resumed the debate documenting in Wfs1−/− mice disturbed mitochondrial network dynamics and activation of mitophagy^13^. Noticeably, recessive mutations in CDGSH Iron Sulfur Domain 2 (*CISD2*) gene, encoding a protein localized in the mitochondria-associated ER membranes (MAMs), are causative for Wolfram syndrome type 2^14–19^. As shown for Wfs1, also Cisd2 knock out mouse model and mouse embryonic fibroblast (MEF) studies documented mitochondrial dysfunction as evidenced by ultrastructural alterations of mitochondria, increased oxygen consumption and ADP/ATP ratio, and overload of mitochondrial calcium (Ca^2+^)^20,21^. Moreover, in patient-derived fibroblasts carrying a *CISD2* mutation it has been reported enhanced Ca^2+^ efflux from mitochondria to ER and a mitochondrial bioenergetics defect^17^.

WS is typically characterized by severe optic atrophy with variable degrees of visual loss and visual field defects^22^. OCT studies documented in some case series a preferential involvement of the inferior sector of the optic nerve^22^, but one post-mortem study reported a prominent loss of retinal ganglion cell (RGC) axons in the temporal sector, implicating loss of the papillomacular bundle (PMB), the hallmark of mitochondrial optic neuropathies such as Leber’s Hereditary Optic Neuropathy (LHON) and Dominant Optic Atrophy (DOA)^23^. Similar to DOA, *WFS1* heterozygous mutations have been recently associated with dominant segregation of optic atrophy and deafness^24^.

We thoroughly investigated a cohort of 13 recessive WS adult cases by extensive neuro-ophthalmological and clinical evaluations. We revisited the issue of mitochondrial dysfunction by assessing venous lactic acid after standardized exercise, evaluating brain and skeletal muscle energy metabolism *in vivo* by proton and phosphorus MR spectroscopy (^1^H-MRS, ^31^P-MRS), and studying mitochondrial bioenergetics, network morphology and calcium handling in patient-derived fibroblasts.

## Results

### Clinical and genetic findings in WS patients

Mean age for WS patients at our observation was 32.9 ± 13.2 years (n = 13). Average age at onset of visual loss was 10 ± 4 years. Of the 13 subjects, 11 had diabetes mellitus with a mean age at onset of 15.5 ± 12.7 years (7/11 treated with insulin); 2 patients had diabetes insipidus and 6 had hearing loss. Urinary dysfunction was present in 7 subjects and psychiatric symptoms in 6. Five patients had a positive family history for WS. Two patients had consanguineous parents (first degree cousins).

The main clinical findings are summarized in Table 1.

**Table 1:** Clinical findings in WS cohort.

ID	Mutation	Effect	Age	DM onset	DM Therapy	HbA1c (mmol/ mol)	Diabetes insipidus	Hearing loss	Psychiatric symptoms	Urinary symptoms	Other symptoms	LA basal, a.e. and a.r. (mg/dl)	Muscle biopsy
1	c.1381 A > C c.1675G > A	p.T461P p.A559T	28	3	insulin	47	−	+	+	+	none	8,5-**49**-**33** **(2015)** 5.5-**55.5**-**32.5**(**2017**)	NA
2	c.409_426dup16 c.2104 G > A	p.V142fsX251 p.G702S	25	10	insulin	71	−	−	−	−	migraine early puberty	11-17-19	NA
3	c.2002C > T c.2126 T > G	p.Q668X p.V709G	42	13	insulin	56	−	+	−	+	central apnea dysphagia	15-21-15	NA
4	c.977 C > T (homo)	p.A326V	56	15	insulin	60	−	+	+	−	fallot tetralogy bicameral PM	13.4-**37.8**-26.2	normal
5	c.605 A > G (homo)	p.E202G	22	10	diet	42	−	−	−	+	none	8-**35**-17	NA
6	c.1369 A > G c.2104 G > A	p.R457G p.G702S	24	19	insulin	<42	−	−	−	+	none	10,5-**38**-**26**	NA
7	c.1928T > G c.2194 C > T	p.L543R p.R732C	47			38	−	−	+	−	hypertension	15-**32**-22	NA
8	c.1928T > G c.2194 C > T	p.L543R p.R732C	41	29	oral	NA	−	+	−	−	glaucoma	NA	NA
9	c.2206 G > A (homo)	p.G736S	37	11	oral	NA	+	−	+	+	none	13-**53**-**43**	NA
10	c.1552 A > C c.2084 G > T	p.T471P p.G695V	50	47	oral	NA	+	+	+	+	dementia dysphagia ataxia	5.5-**32.4**-19	Mild signs of mitochondrial myopathy
11	c.2104 G > A c.2453_66dup14	p.G702S p.F724SfsX766	18	12	insulin	NA	−	−	NA	NA	none	9.3-8.4-17.7-11.9	NA
12	c. 1553 T > A (homo)	p.M518K	16			36	−	−	−	−	none	9-**54**-**32** (**2014**) 7.1-6.6-**31.5**-14.7 (2017)	normal
13	c.387 G > A (homo)	p.W189X	22	2	insulin	67	−	+	+	+	hypothiroidism	8.1-8.9-9.2-6.5	NA

The table shows for each patient genetic and clinical findings. Available muscle biopsies and laboratory results (HbA1c and LA levels after excercise) are also shown. a.e. after exercise; a.r. after recovery. In bold and underlined abnormal LA values (normal values 5–22 mg/dL). Hom: homozygous, DM: diabetes mellitus, NA not available, LA: lactic acid.

Genetic analysis for mutations in *WSF1* gene revealed compound heterozygous mutations in 8 subjects (61%), the remaining 5 carried homozygous mutations (38%) (Table 1).

Neuro-ophthalmological findings are summarized in Table 2.

**Table 2:** Neuro-ophthalmological results in WS cohort.

ID	Age	Visual loss onset	VA (OD	VA OS	Pupils	Ishihara Test	FOO Optic disc pallor	FOO pigmentary changes	FOO others	VF Defect	VF MD (dB) OD	VF MD (dB) OS	VF Fovea OD	VF Fovea OS
1	28	10	0,013	0,01	sluggish (L > R)	0/12	diffuse	mild peripheral	tilted disk	diffuse	−33,76	−33,71	NA	NA
2	25	17	0,32	0,4	sluggish (R > L)	0/12	**temporal (R > L)**	none	Retinal drusen	diffuse	−3,37	−2.76	33	31
3	42	10	0,08	0,025	miotic; no response	0/12	diffuse	macular dystrophy OD	small disc	diffuse	−19,96	−22,61	24	15
4	56	6	0,02	0,01	sluggish (L > R)	0/12	diffuse (**>T**)	none	diabetic retinopathy; macular drusen	diffuse	−31,04	−30,59	NA	NA
5	22	10	0,16	0,25	sluggish	NA	**temporal**	mild perifoveal salt-pepper	none	**central scotoma**	−6,17	−4.36	23	27
6	24	6	0,4	0,63	sluggish (R > L)	0/12	diffuse (**>T**)	none	none	diffuse	−4.37	−5,94	31	35
7	47	6	0,5	0,4	sluggish (R > L)	0/12	diffuse (**>T**)	none	small disc, temporal excavation	diffuse	−5,51	−6,74	33	31
8	41	17	0,125	0,16	sluggish (L > R)	0/12	diffuse	none	peripheral retinal drusen	diffuse	−5.89	−2.27	27	22
9	37	11	0,063	0,063	no response (L > R)	0/12	**temporal (R > L)**	none	none	diffuse	−18.80	−24.09	26	20
10	50	6	0,02	0,06	sluggish (R > L)	NA	diffuse (**>T**)	NA	none	**central scotoma**	NA	NA	NA	NA
11	18	12	0,32	0,25	normal	NA	diffuse	none	none	diffuse	−6.25	−9.07	NA	NA
12	16	13	0,4	0,32	normal	0/12	diffuse (**>T**)	none	perifoveal drusen, retinal cysts (L > R)	diffuse	−6.15	−5.60	33	32
13	22	6	0.16	0.013	no response	0/12	diffuse	none	none	diffuse	−30.20	−34.08	19	16

VA: visual acuity; OD: right eye; OS: left eye; R: right; L: left; T: temporal; BOO: biometry, FOO: fundus; ON: optic nerve; VF: visual fields; MD: mean deviation, NA: not available.

**Figure 1:**
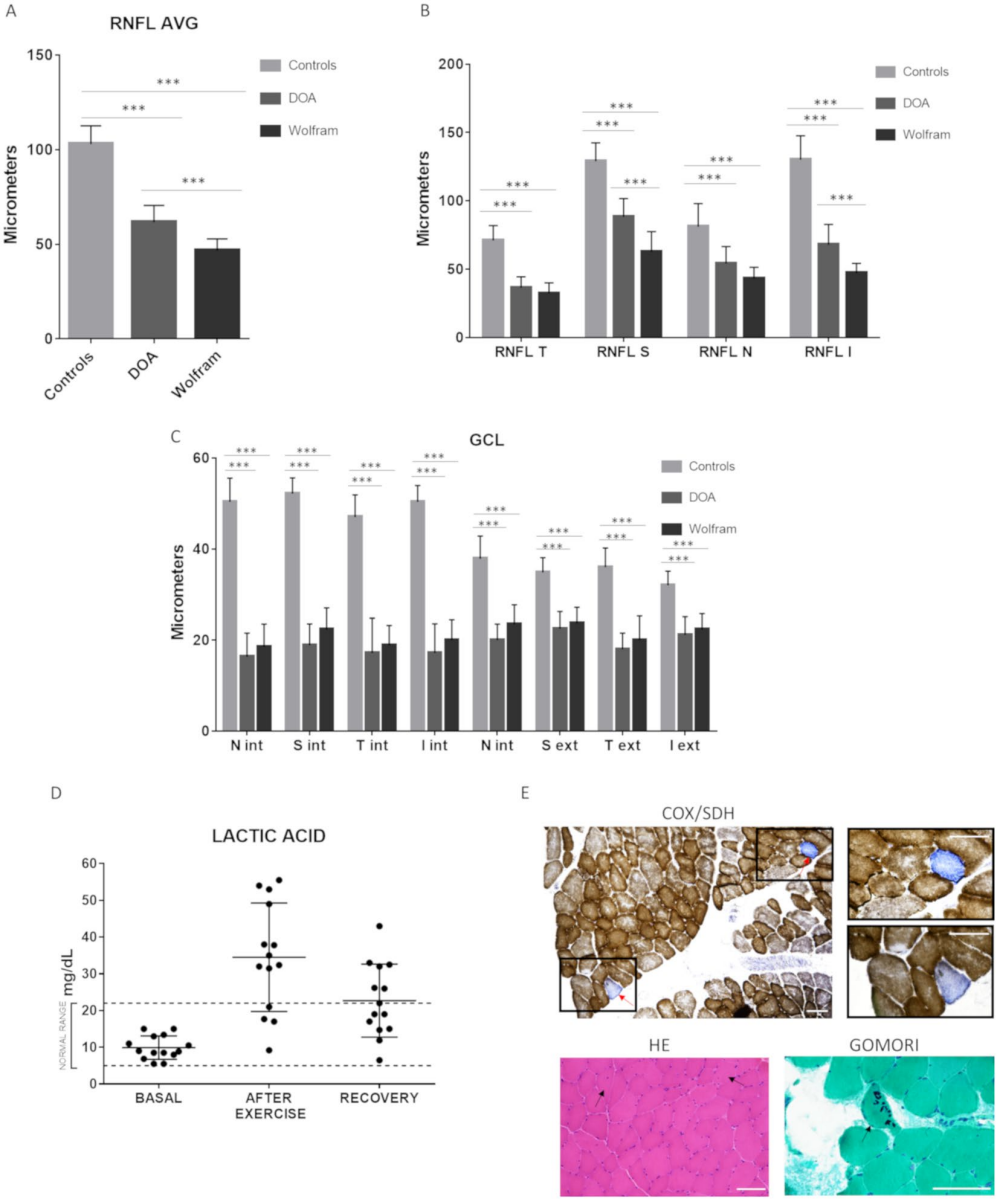
OCT, lactic acid and skeletal muscle results. (**A**,**B**) Panel A and Panel B show, respectively, average and single quadrant RNFL thickness in the tree groups (Controls, DOA, WS). ^***^p < 0. 001 *one-way ANOVA test*. (**C**) Panel C shows sectorial GCL thickness.RNFL: retinal nerve fiber layer; AVG: average; T: temporal; S: superior; N: nasal; I: inferior; int: internal; ext: external; GCL: ganglion cells layer; DOA: dominant optic atrophy; WS: Wolfram syndrome. ^***^p < 0. 001 *one-way ANOVA test*. (**D**) Lactic acid measured in blood at baseline, after exercise and after recovery time in 12 WS patients. Normal values: 5–22 mg/dL. (**E**) COX/SDH, HE and Gomori modified trichrome staining of the muscle biopsy from patient n. 10. Two COX negative fibers (blue fibers highlighted in the insets), two fibers with central nuclei and one fiber with cytoplasmic bodies are pointed by arrows. Bars: 100 µm.

Mean visual acuity was 0.20 ± 0.18 with impaired color vision in all cases, and abnormal pupillary response in 11/13 (84%) patients. Fundus oculi was abnormal in all patients demonstrating diffuse optic nerve pallor in 10/13 (77%) (more temporal in 5/10) and temporal pallor only in 3/13 (23%). Visual fields demonstrated a generalized defect in 11/13 (84%) and central scotoma in 2/13 (15%). OCT demonstrated diffuse and severe retinal nerve fiber thinning in all cases examined. Average retinal nerve fibre layer (RNFL) thickness was 46.6 ± 6.1 micrometers (Fig. 1A). Quadrant RNFL thinning was consistent with fundus examination with the temporal sectors showing the lowest thickness (32.3 ± 7.6), followed by nasal (43 ± 8.3), inferior (47.08 ± 7.06) and superior quadrants (62.7 ± 14.6) (Fig. 1B). Furthermore, we compared RNFL thickness data of WS patients to a group of 70 healthy age and sex-matched controls and 16 DOA patients carrying Optic Atrophy 1 (*OPA1*) mutations (controls 32.1 ± 10.8, DOA 31.43 ± 14.1, WS 31.5 ± 12.7 years of age) (Fig. 1A,B). The average and quadrants RNFL thickness results and comparisons between groups are shown in Fig. 1A,B. Statistical analysis with one-way ANOVA demonstrated a significant average RNFL thinning in both DOA and WS patients compared to controls (p < 0.001) (Fig. 1A). The average RNFL thickness was lower in WS compared to DOA (p < 0.001) (Fig. 1A), at difference of visual acuity (VA), which was not significantly different between groups (p = 0.9) (Table 2). Considering separately the four optic disc quadrants, one-way ANOVA with Bonferroni correction demonstrated a significant difference between groups for all quadrants, except for temporal and nasal quadrants, which resulted not significantly different in WS compared to DOA. The thickness of the four optic disc quadrants was significantly thinner in both WS and DOA compared to controls (p < 0. 001) (Fig. 1B).

The results on segmentation analysis (ganglion cell layer thickness) for the 9 sectors included are shown in Fig. 1C. One-way ANOVA with Bonferroni correction showed that the ganglion cell layer (GCL) thickness was significantly reduced in both WS and DOA compared to controls for all nine sectors (p < 0.001), whereas the difference between WS and DOA was not statistically significant for all sectors.

### Lactic acid evaluation

Basal lactic acid values were normal in all patients assessed (12/13). Lactic acid after exercise was abnormal in 8/12 (66%) patients and in 4/8 (50%) remained abnormally elevated also after the recovery time (Table 1 and Fig. 1D).

### Muscle biopsy

Muscle biopsy was performed in 3 patients (n. 4, n. 10, and n. 12 in Table 1). Muscle biopsy from patient 10 showed not specific alterations, such as mild variability of fiber size, some central nuclei, a few scattered cytochrome c oxidase (COX) negative fibers and an irregular distribution of oxidative activity with some fibers showing increased subsarcolemmal succinate dehydrogenase (SDH) staining; in one single fiber multiple cytoplasmic bodies were observed with the Gomori trichrome staining (Fig. 1E). This patient presented the most severe phenotype of the WS cases investigated (see Table 1). Muscle biopsy from patient n. 4 showed not specific changes with scattered atrophic fibers and rare central nuclei; cytoplasmic or subsarcolemmal rimmed vacuoles were observed in some muscle fibers; the staining for oxidative activity showed only a mild increase of subsarcolemmal SDH staining in a few fibers, whereas COX negative fibers were absent (Fig. S1). Muscle biopsy from patient n.12 was normal (Fig. S1).

### Brain magnetic resonance imaging and magnetic resonance spectrometry findings

Conventional brain Magnetic Resonance Imaging (MRI) findings are reported in Table 3. In all patients, brain MRI findings demonstrated atrophy of brainstem, with a T1 hypointensity in the ventral pons and thinning of bilateral anterior optic pathway. Increased signal intensity in the bilateral peritrigonal areas/optic tracts was observed in 5/10 patients in T2 weighted images. In all patients, the neurohypophyseal “bright signal” on T1-weigheted images was present.

**Table 3:** Conventional Brain MRI findings in Wolfram syndrome patients.

Case	Sovratentorial Brain				Infratentorial Brain				
	Atrophy		Signal alterations	Neuro-Hypophyseal “bright signal”	Atrophy				Signal alterations
	Cerebral	Anterior Optic Pathway	White Matter (T2 hyperintensity & T1 hypointensity)		Mesencephalon	Pons	Medulla allungata	Cerebellum	Pons
Case 1 (CA)	parietal cortex III ventricle enlargement	severe	peritrigonal/optic radiations	present	moderate	severe	moderate	moderate	T_1_ hypointensity
Case 2 (BM)	/	moderate	optic radiations	present	/	mild	/	/	T_1_ hypointensity
Case 3 (AH)	/	mild	/	present	/	mild	/	/	T_1_ hypointensity
Case 4 (DEF)	/	mild	/	present	/	moderate	/	/	T_1_ hypointensity
Case 5 (VS)	parieto-occipital cortex III ventricle enlargement	moderate	peritrigonal/optic radiations	present	mild	severe	moderate	moderate	T_1_ hypointensity
Case 6 (MT)	/	mild	/	present	/	mild	/	/	T_1_ hypointensity
Case 7 (BL)	mild III ventricle enlargement	moderate	peritrigonal/subcortical	present	/	moderate	/	/	T_1_ hypointensity
Case 8 (ME)	/	severe	peritrigonal/optic radiations	present	/	mild	/	mild	T_1_ hypointensity
Case 9 (BS)	mild III ventricle enlargement	severe	/	present	/	moderate	mild	/	T_1_ hypointensity
Case 10 (RS)	parieto-occipital cortex	moderate	/	present	/	moderate	mild	mild	T_1_ hypointensity

/= absent.

**Figure 2:**
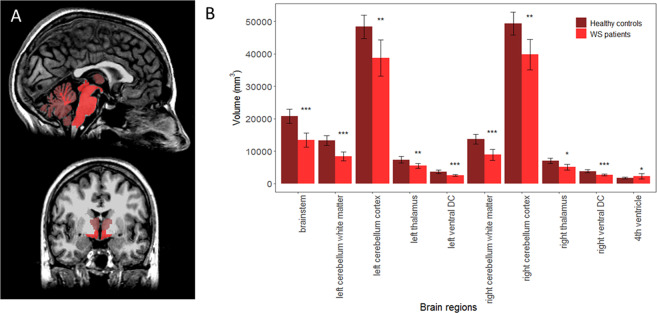
Regional Subcortical Volumes: WS patients *vs* healthy controls. (**A**) The brain regions which resulted atrophic in WS patients compared to healthy controls are highlighted on sagittal and coronal views from the T1 image of a representative WS patient (dorsal and ventral pons, midbrain, cerebellar white matter and diencefalic region, p < 0.001, light red, and cerebellar cortices and bilateral thalami with a lesser degree of atrophy, p < 0.05, dark red). Images are shown in radiological convention (left is right). (**B**) Significant results (*p < 0.05, **p < 0.01, ***p < 0.001) of the comparisons are reported in the bar plot. For a complete overview of p values, averages and multiple comparisons corrections see Table S2. DC: Diencephalon; WS: Wolfram syndrome.

Voxel-based morphometry (VBM) showed cortical grey matter volume loss in the calcarine (p < 0.05) and cerebellar cortices (p < 0.001), specifically in the bilateral I-IV, V, VI, VIIb, and left VIIIa; bilateral crus I, II; and vermis VI, VIIIa, IX (Fig. S2 and Table S1). FreeSurfer analysis showed lower total intracranial volume (TIV) (p = 0.018) and lower volume of ventral diencephalon, brainstem and cerebellar white matter in WS patients (p < 0.001) (Fig. 2 and Table S2).

Tract-Based Spatial Statistics (TBSS) highlighted lower fractional anisotropy (FA) (driven by radial diffusivity increase) for patients mostly within optic radiations and optic tracts, posterior limbs of internal capsules, cerebellar peduncles (inferior, middle and superior) and to less extent corpus callosum (p < 0.05, corrected) (Fig. S2 and Table S1).

WS patients had lower cerebellar hemisphere N-acetyl-aspartate/Creatine (NAA/Cr) (p = 0.003) and lower N-acetyl-aspartate/myoinositol (NAA/mI) (p < 0.001) and higher mI/Cr (p = 0.012) in comparison to controls. Lower NAA/Cr (p = 0.004) and NAA/mI (p = 0.005) were found in the ventral pons, while no differences were found in parieto-occipital white matter (POWM) (Table S3).

^1^H-MRS failed to demonstrate, likewise in healthy controls, pathological accumulation of lactate in lateral ventricles in all WS patients. ^31^P-MRS muscle metrics showed no differences between patients and controls at rest, during exercise and the post-exercise recovery (Table 4). In particular, WS patients showed normal post-exercise indices of muscle oxidative metabolism such as time constant (TC) of phosphocreatine (PCr) resynthesis and maximum rate of mitochondrial ATP synthesis.

**Table 4:** Skeletal muscle ^31^P MR Spectroscopy results.

	Variable	WS (n = 8, age = 31 ± 13 y, 2 M)		Controls (n = 8, age = 32 ± 11 y, 4 M)		Mann-Whitney Test
		mean	(SD)	mean	(SD)	p value
Rest	**pH**	7.02	0.02	7.03	0.002	0.20
	**PCr/Pi**	8.05	1.31	7.56	1.24	0.51
		**WS** **(n = 6, age 33 ± 14 y, 1 M)**		**Controls** **(n = 6, age = 32 ± 11 y, 2 M)**		
Exercise	**Duration (min)**	4.34	2.92	4.82	2.40	1
**End-exercise**	**pH**	6.91	0.11	6.85	0.11	0.31
	**[PCr] %**	40.1	11.7	41.5	11.0	0.70
Post-exercise recovery	**TC PCr (sec)**	39.44	7.99	35.33	9.16	0.59
	**V**_**max**_ **(mM/min)**	54.71	12.15	52.56	12.54	0.82

Skeletal muscle ^31^P MR Spectroscopy results at rest, during aerobic exercise and in the post-exercise recovery in WS patients (6/8 performed muscle exercise) and matched healthy controls (n = 8). Data is reported as mean ± SD years; pH= cytosolic pH; PCr, phosphocreatine; Pi, inorganic phosphate; [PCr] %= PCr concentration at the end of exercise expressed as percentage of the concentration at rest; TC PCr: time constant of PCr resynthesis; V_max_: maximum rate of mitochondrial ATP synthesis.

None of the MRI metrics showed significant correlation with the age of patients, with exception of the NAA/Cr ratio in the cerebellar hemisphere, which showed a strong correlation with age in WS patients (rho = −0.817, p = 0.007), but not in healthy controls (rho = 0.006; p = 0.987).

### Fibroblast cell studies

Fibroblasts derived from two WS patients were studied to evaluate WFS1 protein expression, mitochondrial bioenergetics, mitochondrial network morphology and intracellular Ca^2+^ dynamics.

**Figure 3:**
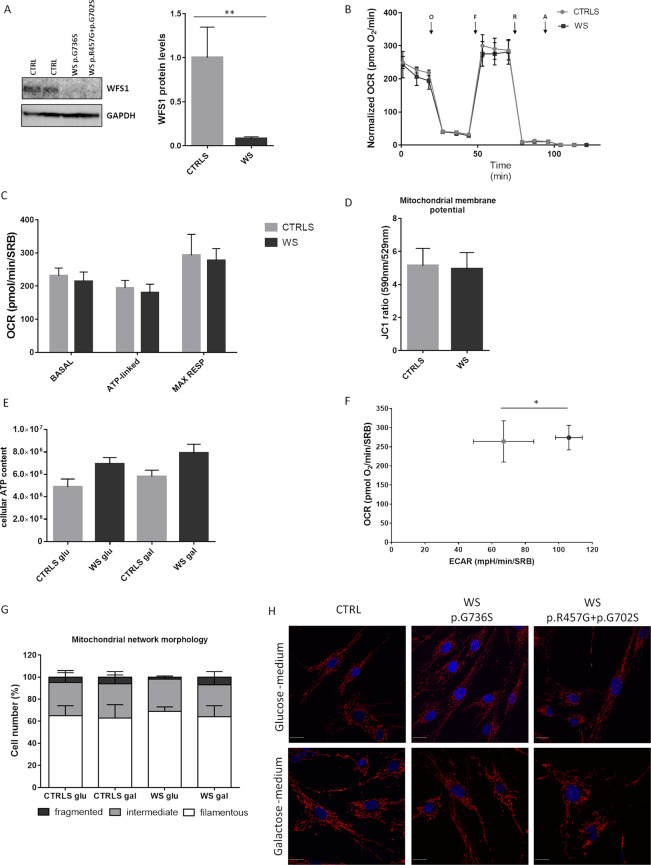
Mitochondrial capacity in Wolfram fibroblasts. (**A**) WFS1 quantification by Western blot. A representative image grouping different parts of the same blot (original images in Supplementary information file) and densitometric analysis are shown. Error bars, s.d. of three biological replicates. **p < 0.01 *upaired t-test*. (**B**) Oxygen consumption rate (OCR) traces of control and Wolfram fibroblasts, expressed as pmol O_2_/min, in basal conditions and after the injection of oligomycin (O), carbonyl cyanide 4-(trifluoromethoxy)phenylhydrazone (FCCP; F), rotenone (R) and antimycin A (AA). OCR values are normalized on protein content (SRB). Error bars, s.d. of two independent experiments for each cell line. (**C**) Basal, ATP**-**linked and maximal respiration were calculated from OCR traces and are reported in the graph as means ± s.d. Differences are not significant based on the *two-way ANOVA test*. (**D**) Mitochondrial membrane potential expressed as JC-1 fluorescences ratio (590 nm/529 nm). Error bars, s.d. of four independent experiments for each cell line. (**E**) Cellular ATP content measured in standard conditions (glucose-medium) and after 48 hours of incubation in a glucose-free medium supplemented with 5 mM galactose. Error bars, s.d. of 2 independent experiments for each cell line. Differences are not significant based on the *two-way ANOVA test*. (**F**) Energetic map reporting basal OCR and ECAR values normalized on protein content (SRB). Error bars, s.d. of two independent experiments for each cell line. *p < 0.05 *unpaired t-test*. (**G**) Classification of cells based on the mitochondrial morphology (filamentous, intermediate and fragmented mitochondria) by blind test. 30–50 cells for each cell line and condition were scored. Data are expressed as mean ± s.d. of three independent experiments for each cell line. (**H**) Mitochondrial network morphology evaluated by confocal microscopy, in glucose-medium and after 48 hours of incubation in the galactose-medium. Mitochondria are stained with MitoTracker Red (red fluorescence) and nuclei are stained with Hoecsht (blue fluorescence), representative images are reported in the figure. White bar, 25 µm. All data are expressed as mean of two control fibroblasts (CTRLS) and two *WFS1* mutant fibroblasts (WS).

WFS1 protein expression was evaluated by Western blot in control and WS fibroblasts. Although both patients carried missense mutations in *WFS1*, we observed a dramatic reduction of WFS1 expression in mutant fibroblasts compared to controls (p = 0.0013) (Fig. 3A), indicating the presence of a loss-of-function mechanism. Oxygen consumption, an indicator of mitochondrial respiratory capacity, resulted comparable in WS and control fibroblasts, both in basal condition and after the addition of oligomycin (ATP-linked respiration) and carbonyl cyanide 4-(trifluoromethoxy) phenylhydrazone (FCCP) (maximal respiration) (Fig. 3B,C). Concordantly, mitochondrial membrane potential was not affected in WS fibroblasts (Fig. 3D). Total ATP was increased, although not reaching a statistical significance, in WS fibroblasts compared to controls, in standard conditions (glucose medium) and when cells were forced to use mitochondria to produce ATP (galactose medium) (Fig. 3E). Moreover, as shown in the energetic map (Fig. 3F), WS and controls cells had similar oxygen consumption rate (OCR) but extracellular acidification rate (ECAR), an indicator of glycolysis, was increased in WS cells compared to controls (p = 0.0168), suggesting a higher contribute of glycolysis rather than mitochondrial respiration to the cellular ATP production. Mitochondrial network morphology was not compromised in patients’ fibroblasts grown in glucose-medium, since the percentage of cells characterized by filamentous, intermediate and fragmented mitochondria was comparable to that of control fibroblasts. After 48 hours of incubation in the galactose-medium, the percentage of cells with fragmented mitochondria was again similar in WS and control fibroblasts, indicating the absence of a mitochondrial network morphology defect, congruent with a normal bioenergetic function (Fig. 3G,H).

**Figure 4:**
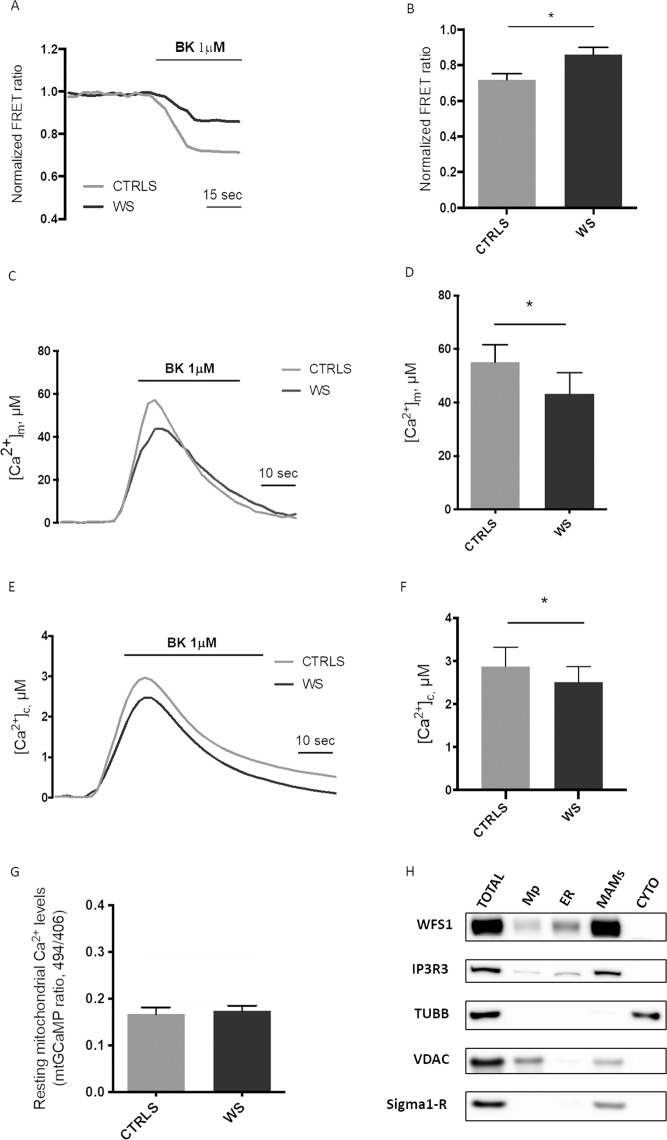
Intracellular Ca^2+^assessment and MAM localization of WFS1. (**A**,**B**) ER Ca^2+^ release induced in fibroblasts from controls and WS patients by stimulation with 1 µM Bradykinin. Cell were transfected with the ER- Ca^2+^ sensor D1ER. Changes in ER-Ca^2+^ release were monitored by evoking Ca^2+^ release via iP3 receptors on the ER. Representative traces (**A**) and graph depicting the FRET ratio (**B**) are shown. Data are expressed as mean ± s.d. of three independent experiments for each cell line. *p < 0.05 *unpaired t-test*. (**C**,**D**) Aequorin-based measurements of mitochondrial Ca^2+^ uptake induced in fibroblasts from controls and WS patients by stimulation with 1 µM Bradykinin. Data are expressed as mean ± s.d. of three independent experiments for each cell line. *p < 0.05 *unpaired t-test*. (**E**,**F**) Aequorin-based measurements of cytosolic Ca^2+^ induced in fibroblasts from controls and WS patients by stimulation with 1 µM Bradykinin. Data are expressed as mean ± s.d. of three independent experiments for each cell line. *p < 0.05 *unpaired t-test*. (**G**) Resting mitochondrial Ca^2+^ levels in control and WS fibroblasts evaluated by using ratiometric imaging of the mitochondrial targeted GCaMP. Data are expressed as mean ± s.d. of three independent experiments for each cell line. (**H**) Western blot of subcellular fractions isolated from A549 cells, where IP3R3 was used as an ER marker, Sigma 1-R as a MAM marker, VDAC as a mitochondria marker and β-Tubulin as cytosolic marker. TOTAL, total lysate; Mp, pure mitochondria; ER, endoplasmic reticulum; MAMs, mitochondria-associated membranes; CYTO, cytosol. Image is generated by grouping different parts of the same blot (original images in Supplementary information file).

Different reports indicate that mitochondrial metabolism and ATP production are both mechanisms finely tuned by Ca^2+^ dynamics, in particular by a proper ER-mitochondrial Ca^2+^ transfer^25^. When the ER-Ca^2+^ release is evoked, Ca^2+^ can be released directly into cytosol or taken up by mitochondria. Increases in this intra-mitochondrial Ca^2+^ levels, in turn, stimulates ATP production throughout activation of Ca^2+^-sensitive dehydrogenases belonging to the Krebs cycle. Hence, we decided to investigate Ca^2+^ handling, implicating the ER, mitochondrial and cytosol compartments, by measuring agonist dependent Ca^2+^ transients in healthy control and Wolfram fibroblasts. Cells were transfected with ER targeted cameleon (D1ER), a fluorescent probe that monitors ER-Ca^2+^ concentration variations, and then stimulated with the agonist bradykinin (BK), which induces ER Ca^2+^ release through the IP3Rs. We found that WS fibroblasts released lower amounts of Ca^2+^ from the ER, than control samples (Fig. 4A,B). This result suggested that Wolfram cells were characterized by a reduced flux of Ca^2+^ from the ER. As consequence, we found lower Ca^2+^ accumulations in mitochondria (Fig. 4C,D) and cytosol (Fig. 4E,F) in WSF fibroblasts as compared to controls after BK stimulation, without finding any variation in the resting mitochondrial Ca^2+^ levels (Fig. 4G). The correct Ca^2+^ transmission between ER and mitochondria is finely tuned by the contact sites between the organelles, known as MAMs^26^. Different proteins have been found to localize in these fractions and determine positive or negative modulation of Ca^2+^ transmission between ER and mitochondria^27^. Having found an important deregulation in mitochondrial Ca^2+^ accumulation in Wolfram fibroblasts, we asked whether Wolfram would be localized at MAMs site to regulate Ca^2+^ homeostasis. Subcellular fractionation confirmed this hypothesis and revealed that WFS1 was present in considerable amounts in the MAMs compartment (Fig. 4H).

## Discussion

Our study, by using multiple *in vivo* and *in vitro* approaches, demonstrates the absence of a primary mitochondrial dysfunction in WS patients. However, in specific tissue or cell types characterized by high energy requirements (i.e neuronal cells), a secondary metabolic dysfunction may occur likely mediated by calcium mishandling between ER and mitochondria. In support of this interpretation, we documented that WFS1 is enriched at MAMs, and the virtual absence of WFS1 protein in WS patients, as also reported by others^28^, most probably leads to the documented defective calcium efflux from ER.

WS is a neurodegenerative disorder typically characterized by the occurrence of optic atrophy and diabetes in childhood, frequently with additional symptoms, such as diabetes insipidus, deafness, and psychiatric disorders^29^. Given the close similarity of WS with mitochondrial disorders, we thoroughly investigated the occurrence of mitochondrial abnormalities in WS patients. Lactic acid evaluation after standardized exercise showed abnormally increased lactic acid only after exercise in most patients. However, a confounding factor impinging on the evaluation of this parameter is the concomitant presence of diabetes in these patients, which may result in abnormal increase of lactic acid, as previously reported^30^, indicative of enhanced non-oxidative glycolysis leading to lactate accumulation^31^.

Considering the ophthalmological phenotype of our WS patients, we found a pattern of diffuse optic atrophy, not strictly selective for the temporal fibers of the papillomacular bundle, which is instead typical for DOA, a disease with a primary mitochondrial dysfunction^32^. Overall, WS patients showed a more severe RNFL thinning at OCT, significant in comparison to DOA patients for all sectors except for the nasal and temporal quadrants. Interestingly, the significant loss of retinal nerve fibers in WS does not translate into a significant reduction of visual acuity compared to DOA, pointing to a more generalized loss of fibers in the optic nerve, not specific for the papillo-macular bundle, at difference with mitochondrial optic neuropathies^33^.

Visual field (VF) demonstrated the presence of a central scotoma only in 2 WS cases, whereas for other WS patients visual field defects were heterogenous, again not suggesting a classical mitochondrial pattern^34^. The neuro-ophthalmological phenotype has been poorly defined in WS until recently^35–37^. Grenier and colleagues^22^ evaluated OCT findings in 15 WS cases. Eleven patients carried autosomal recessive (ar)-WS mutations (8 with diabetes and 3 without diabetes) and four patients carried autosomal dominant (ad)-WS mutations in association with hearing loss. The authors reported with spectral domain OCT a more profound RNFL thinning in syndromic ar-WS than in WS cases without diabetes, especially in the inferior sector of the optic nerve. Time-domain OCT demonstrated a more severe RNFL thinning in the ar-WS group in both the superior and inferior quadrants compared with ad-WS cases^22^. Another study investigated OCT findings in 12 genetically-confirmed WS children. WS patients showed a greater RNFL thinning especially in the superior and inferior sectors and in the brain MRI visual pathway measurements compared with individuals with type 1 diabetes and controls. Interestingly, the thickness of the intraorbital parts of the optic nerves evaluated by brain MRI correlated with the superior RNFL thickness of the optic nerve^37^. Moreover, OCT demonstrated optic nerve thinning in a group of WS patients followed over a period of about 2 years^36^. Further studies are needed to firmly consolidate the neuro-ophthalmological features of WS and their natural history progression.

Brain MRI findings have been previously reported in WS patients^38–40^ and atrophy of the cerebellar cortices and brainstem, specifically in the pons, are well-documented features. Interestingly, at visual inspection, we observed a pathological dilatation of the inferior aspect of the third ventricle in four patients, two of them with diabetes insipidus, confirmed by a FreeSurfer group analysis detection of high - significant volume reduction in the ventral diencephalic region. These results are consistent with the postmortem study of one WS patient showing a specific neuronal loss in paraventricular and supraoptic nuclei in the hypothalamus^41^. We expanded the findings of the cerebellar atrophy reporting a significant age-related NAA/Cr loss in the cerebellar hemisphere, revealed with a single voxel 1H-MRS, suggesting a degenerative neurometabolic process at this level, which evolves with age. The atrophy of the ventral pons was associated in all patients with T1 hypointensity present in pons in 3D fast spoiled gradient echo (FSPGR) images. This patchy MRI signal abnormality in the ventral pons is consistent with a previous neuropathological study of a patient showing a patchy pattern of loss in the pontine base, in which areas of gliosis alternated to areas in which neurons are spared, with no preferential involvement of a specific area^41^. In the sovratentorial brain, we confirmed diffuse white matter changes without significant NAA/Cr loss or correlations with the age of patients, suggesting a primary neuropathological feature of Wolfram Syndrome, as previously reported^40^. MRS studies clearly failed to demonstrate the presence of brain and skeletal muscle oxidative metabolism in WS patients at different stages of disease, in contrast with previous findings in patients with mitochondrial optic neuropathies such as LHON and DOA (*OPA1* mutations)^42,43^. The failure of documenting a primary defect of skeletal muscle oxidative metabolism reinforces the interpretation that increase of lactic acid after standardized effort is ascribed to the concurrent diabetes. In addition, the analysis of three muscle biopsies from our patients were not informative for a clear-cut primary mitochondrial dysfunction, in line with a previous report^44^.

Similarly to MRS, fibroblast studies did not support the presence of defective mitochondrial bioenergetics and network morphology, differently from previous reports^13^. Concordantly with our results, a recent study failed to evidence any defect in mitochondrial morphology or mitochondrial membrane potential in fibroblasts from WS patients carrying loss-of-function mutations in *WFS1*^28^. On the other hand, in this study WS fibroblasts showed impaired OCR and activity of complexes I and IV, possibly related to a reduced expression of the neuronal calcium sensor 1 (NCS1), an interactor of WFS1^28^. The discrepancy between our and these previous findings can be referred to the different models used, i.e. neuronal cells from knockout mice^13^, or to the inter-individual phenotypic expression in WS patients and consequently in fibroblast cell lines, as already reported for *OPA1* mutations^45^.

Interestingly, the tendency of increased cellular ATP content and the significantly higher ECAR, support the hypothesis of increased non-oxidative glycolysis, a possible effect of dysregulated glucose metabolism due to diabetes^31^.

Moreover, after stimulation with bradykinin, we observed an altered mitochondrial Ca^2+^ uptake driven by a reduced Ca^2+^ release from ER. Remarkably, we did not find variations in Ca^2+^ basal amounts, suggesting that the alteration in [Ca^2+^]_m_ dynamics were not due to a general perturbation of the mitochondrial compartment. Supportive to this, changes were only found when we analyzed the driving force necessary for the passive Ca^2+^ entry into mitochondria. In addition, the possibility that decreases in [Ca^2+^]_m_ uptake of patient fibroblasts were due to decreased ER calcium release was further confirmed by observing parallel reductions in cytosolic Ca^2+^ in response to IP3-evoked Ca^2+^ release.

An appropriated ER-mitochondrial Ca^2+^ transfer, followed by proper mitochondrial Ca^2+^ accumulation, is critical for numerous physiological processes and modulates the activity of several mitochondrial enzymes belonging to the Krebs cycle, because several dehydrogenases of the tricarboxylic acid cycle require Ca^2+^ to preserve the functioning of mitochondrial electron chain members. Among them, pyruvate dehydrogenase phosphatase 1 (PDP1) regulates the activity of pyruvate dehydrogenase, thus determining the metabolic fate of pyruvate^46^. Therefore, low levels of mitochondrial calcium uptake might also result in decreased activity of pyruvate dehydrogenase, leading to increased pyruvate conversion into lactate (non-oxidative glycolysis). WFS1 has been previously related to intracellular Ca^2+^ homeostasis, also in the context of pancreatic β-cells, thus connecting calcium to diabetes^28,47–49^. In support of a role for WFS1 in regulating Ca^2+^ efflux from ER to mitochondria, we provide the first direct evidence by Western blot that WFS1 protein localization is enriched in the MAM fractions, confirming previous indications from proteomic analysis^50,51^.

Our study, by tackling at clinical, biochemical, neuro-radiological and cellular levels the issue of mitochondrial dysfunction in WS, contributes substantially to clarify that mitochondrial dysfunction is not a primary event in the pathogenic mechanism, but may occur secondarily as a byproduct of calcium mishandling. The key interaction, in fact, is possibly localized at the MAMs, where we found that Wolframin is enriched. Further studies are warranted to mechanistically define the ER-mitochondrial calcium handling in the pathogenesis of WS. Calcium mishandling and ER-stress may have far reaching consequences in the context of neuronal cells^19^, representing a druggable target for therapeutic strategies in WS patients, as suggested by *WFS1* knock-out cell models^47,52^.

## Methods

### Ethical approval and consent to participate

All the procedures on patients were conducted according to the Declaration of Helsinki and after having obtained the approval of the Independent Ethical Committee “Area Vasta Emilia Centro CE-AVEC” (CE 211/2018/SPER/AUSLBO). Informed written consent for study participation have been obtained from patients and volunteers.

### Patient cohort

We investigated a cohort of 13 WS adult cases (5 males) carrying recessive mutations in *WFS1* gene. Clinical information included family history, onset of OA and DM. Other cardinal symptoms of WS, concomitant pathologies and therapies were also collected.

### Genetic testing

Genetic testing was performed by direct sequencing of the *WFS1* gene^53^, or by a next generation sequencing based (Illumina, TruSeq Custom Amplicon) diagnostic panel dedicated to hereditary optic neuropathies, including *WFS1*.

### Neuro-ophthalmological assessment

Neuro-ophthalmological examination included visual acuity, color vision (Ishihara test), pupil light reflex, slit lamp examination and fundus picture. Assessment of the anterior visual pathways was performed through VF (Humphrey Field Analyzer, protocol Sita Standard 30-2, Zeiss, San Leandro, CA, USA) and OCT. OCT protocol included the evaluation of RNFL thickness (Stratus 3.0, Carl Zeiss Ophthalmic Systems Inc., Humphrey Division, Dublin, CA) according to the RNFL thickness 3.4 acquisition protocol, as previously described^54^ in 12/13 patients. We also performed and segmentation analysis of the retina^55^, including the GCL thickness (Heidelberg Spectralis HRA2, version 6.3.2.0, Heidelberg Engineering, Heidelberg, Germany) in a subgroup of 6/13 WS cases.

OCT data were compared to a control age and sex-matched group (n = 70 for RNFL thickness and n = 6 for GCL analyses) and to a pathological control groups composed by *OPA1*-mutated DOA patients (n = 16 for RNFL thickness and n = 6 for GCL analyses). DOA and WS patients were comparable in terms of visual acuity (DOA 0.24 ± 0.17, WS 0.22 ± 0.21).

At the beginning of the examination, lenses were adjusted for the patient refractive error. Polarization was optimized to maximize the reflective signal, and the best centration of the scan with respect to the optic disk was used. For the statistical analysis, 1 eye was randomly chosen, after verification that there was no statistical difference between the 2 eyes, as previously reported^54^.

### Lactic acid after standardized effort test

We evaluated lactic acid at baseline and after standardized exercise, as previously reported^56^ in 12/13 WS patients. Lactic acid plasma levels of WS patients were compared to the normative data provided by our local laboratory.

### Muscle biopsy

Three WS patients underwent muscle biopsy. Standard stains and histoenzymatic activities were carried out on liquid nitrogen cooled isopentane frozen specimens, as previously described^57^.

### Brain MRI/^1^H-MRS and skeletal muscle ^31^P-MRS acquisitions

All MRI studies were performed in a 1.5 T scanner.

Ten WS patients (3 M, mean age 29 ± 12 years) performed brain MR using a quadrature birdcage head coil. The acquisition protocol included the following sequences: volumetric FSPGR-T1 (voxel = 1 mm^3^), axial FLAIR-T2, coronal FSE T2, diffusion weighted imaging, DWI, (25 gradient directions, b-value = 900 s mm^−2^, voxel=1.25 × 1.25 × 4 mm). Moreover, water-suppressed proton MR spectra were acquired by the point-resolved spectroscopy single-voxel localization sequence (PROBE), as previously reported^58^, in the lateral ventricles in ten WS patients (TR/TE = 1500/288 ms, volume = 3.7–8.2 ml), in the left cerebellar hemisphere in 9 (TR/TE = 4000/35 ms, volume = 6 ml), in the left POWM in 7 (TR/TE = 4000/35 ms, volume = 8 ml) and in the ventral pons in 5 (TR/TE = 1500/40 ms, volume = 1.2–1.5 ml).

Volumetric FSPGR-T1, DWI, and ^1^H-MRS spectra were also acquired in sex- and age-matched healthy subjects (see Additional files 1, 2 and 3, and corresponding legends, for demographic details).

Eight WS patients underwent muscle ^31^P-MRS examination using an 8-cm diameter surface coil placed under the right calf, as previously reported^59^; six of them also performed an aerobic exercise. Not all the patients completed the whole protocol. A group of sex- and age-matched healthy subjects was also studied.

### Brain MRI and ^1^H-MRS analysis

Brain MRI scans were reviewed by two experienced neuroradiologists (RL, LLG) blinded to the clinical data. Results were reported as consensus between the two examiners. Voxel-based morphometry (VBM) analyses were performed with SPM-12.0 supplemented by SUIT, Spatially Unbiased Infratentorial Toolbox^60,61^, to assess voxel-wise cortical gray matter volumes. FreeSurfer was used to determine Total Intracranial Volume (TIV) and extract subcortical region volumes. Diffusion-weighted data underwent standard pre-processing and tensor fitting with FSL (FMRIB Software Library), and TBSS (Tract-Based Spatial Statistics) analysis was also performed. ^1^H-MR spectra were analyzed with LCModel version 6.3.

### Muscle ^31^P-MRS analysis

The fitting software AMARES/MRUI (http://www.jmrui.eu/) was used to estimate peak integrals for PCr, inorganic phosphate (Pi), and the β phosphate of ATP.

Concentrations of metabolites, cytosolic pH, rate of PCr post-exercise recovery (expressed as time constant, TC) and maximum rate of mitochondrial ATP synthesis (Vmax) were calculated as previously reported^59^.

### Fibroblast cell studies

Fibroblasts from two WS patients carrying respectively the compound heterozygous c.1369 A > G (p.R457G)/c.2104 G > A (p.G702S) (n.6 in Table 1) and homozygous c.2206 G > A (p.G736S) (n.9 in Table 1) *WFS1* mutations and two controls were analyzed for WFS1 protein expression, mitochondrial function and Ca^2+^ efflux. Fibroblasts were established from skin biopsies, after having obtained informed and written consent from all patients and controls for the study and for all procedures. The A549 lung cancer cell line was also used for MAMs isolation.

Cells were grown in high glucose DMEM medium (Gibco) supplemented with 10% fetal bovine serum (FBS) (Gibco), 2mM L-glutamine, 100 U/mL penicillin and 100U/ml streptomycin and maintained at 37 °C in an incubator under a humidified 5% CO_2_ atmosphere.

Total protein lysates were obtained as previously described^62^. Protein samples were separated on precast NuPAGE 4–12% Bis-Tris Glicyne gels (Life Technologies) and then transferred on nitrocellulose membranes, using the XcellSure Lock (Life Technologies) apparatus. After blocking with 5% milk, membranes were blotted with primary antibodies anti-WFS1 (Cell Signaling) 1:1000, anti-GAPDH (Sigma Aldrich) 1:10000, anti-IP3R-3 (BD Biosciences) 1:1000, anti-TUBB (Cell Signaling) 1:1000, anti-VDAC1 (abcam) 1:1000, anti-sigmaR1 (Sigma, HPA018002) 1:1000.

Fluorescent secondary antibodies anti-rabbit or anti-mouse (Licor, 1:5000) were used for immunodetection using the LI-COR Odyssey Fc Dual Mode imager.

Mitochondrial respiration was evaluated measuring OCR and ECAR in live cells by the Seahorse XFe24 Flux Analyzer (Agilent), in basal conditions and after the addition of oligomycin (1uM), FCCP (2uM), rotenone (1uM) and Antimycin A (1uM). OCR and ECAR values were normalized on cell density using the colorimetric sulforodhamine B (SRB) assay. Basal, ATP-linked, and maximal respiration were calculated as previously described^63^.

Mitochondrial membrane potential was evaluated using JC-1 fluorescent probe (Thermo Fischer). Briefly, 50.000 cells were seeded on a 96-well plate (View plate, Perkin Elmer) and the day after treated with JC-1 at 5ug/ml concentration at 37 °C. After 20 minutes, cells were washed with PBS and incubated with high-glucose DMEM without phenol red and the 529 nm and 590 nm fluorescences were acquired in a plate reader (Enspire, Perkin Elmer).

Cellular ATP and mitochondrial network morphology were assessed both in standard conditions (glucose-medium) and after 48 hours of incubation in glucose-free medium supplemented with 5 mM galactose (galactose-medium), to force the mitochondrial respiratory chain. Total ATP was quantified using the ATPlite 1step Luminescence Assay System (Perkin Elmer), following manufacturers’ instructions.

Mitochondrial network morphology was evaluated in living cells, stained with Mito Tracker Red (Thermo Fischer) at 10 nM and Hoechst (Thermo Fischer) at 1ug/ml for 20 minutes at 37 °C. Z-stack (0.8 µm) images were captured with an A1 confocal microscope (Nikon Instruments) using a 60x/1.4-NA oil objective and maximum intensity projections were generated using the NIS-elements software (Nikon Instruments). At least 30 cells for each cell line and condition were evaluated in three independent experiments by blind test and classified into three categories based on the mitochondrial morphology (filamentous, intermediate and fragmented mitochondria)^57^.

ER Ca^2+^ release was performed by using the cameleon (calcium sensor) targeted to ER compartment, D1ER. After 36 h from the transfection, fibroblasts were imaged on a Zeiss Axiovert 200 M microscope with equipped with a C-apochromat 40× oil objective (numerical aperture 1.2). Images were captured by using a cooled CCD camera (Photometrics) controlled by MetaFluor 7.0 software (Universal Imaging). The Fluorescence Resonance Energy Transfer (FRET) signal (yellow fluorescent protein (YFP)/CFP) was normalized and changes in ER Ca^2+^ releases (evoked by the agonist BK) were expressed as the ratio of the emissions at 535 and 470 nm.

To evaluate mitochondrial and cytosolic Ca^2+^ uptake, cells were grown on 13-mm round glass coverslips at 60–70% confluence and transduced with aequorin encoded by adenoviral construct mitochondria-targeted mutated aequorin (mtAEQ) and an adenoviral construct cytosolic targeted aequorin (cytAEQ), respectively. The coverslips with the cells were then incubated with 5 μM coelenterazine for 2 hours in 0.1% FBS medium, and then transferred to the perfusion chamber. All aequorin measurements were performed in Krebs-Ringer buffer (135 mM NaCl, 5 mM KCl, 1 mM MgSO_4_, 0.4 mM KH_2_PO_4_, 5.5 mM glucose, 20 mM HEPES, pH 7.4) supplemented with 1 mM CaCl_2_. Agonists were added to the same medium, as specified in the text. To discharge the remaining aequorin pool, cells were lysed in a hypotonic Ca^2+−^rich solution (10 mM CaCl_2_ in H_2_O) with 100 μM digitonin. The light signal was calibrated into [Ca^2+^] values by an algorithm, as previously described^64^.

To evaluate resting mitochondrial Ca^2+^ levels, CTRL and WS mutant fibroblasts were allowed to grow on 24 mm coverslips and transfected with mtGCaMP6m-encoding plasmid. After 36 hours, coverslips were placed in 1 ml of modified KRB (5.5 mM glucose, 1 mM CaCl_2_) and imaged at the Olympus Cell^R multiple wavelength high-resolution fluorescence microscopy system equipped with a 20x/1.3 N.A. Oil. Basal fluorescence of the mtGcAMP ratio (494/406) was registered for 1 min for each condition.

Cell fractionation was performed in A549 lung cancer cell line, as previously described^65^. Briefly, a cells pellet was suspended in homogenization buffer (225 mM mannitol, 75 mM sucrose, 30 mM Tris-HCl pH 7.4, 0.1 mM ethylene glycol-bis(β-aminoethylether)-N,N,N,N-tetraacetic acid (EGTA), and PMSF (phenylmethylsulfonyl fluoride) and gently disrupted by Dounce homogenization. The homogenate was centrifuged to remove nuclei and unbroken cells, and the resultant supernatant was centrifuged to pellet crude mitochondria. Centrifugation of the supernatant at 100.000 g for 90 min (70-Ti rotor; Beckman) resulted in the isolation of ER (pellet) and cytosolic fraction (supernatant). To collect pure mitochondria, crude mitochondria were resuspend in buffer density gradient buffer (225 mM mannitol, 25 mM HEPES, pH 7.4, 1 mM EGTA, 0.1% BSA, 30% Percoll (v/v). After centrifugation, the mitochondrial fraction was isolated two-thirds of the way down the tube, and the MAMs complex was found directly above the mitochondrial fraction.

### Statistical analysis

For ophthalmological and cell studies all numerical data are expressed as mean ± SD. Welch’s unequal variances t-test was used for statistical analysis, unless otherwise indicated. Differences were considered statistically significant for p < 0.05. Normal distribution was assessed with Kolgomorov-Smirnov test and Bonferroni correction applied for multiple comparisons.

For MR data, group comparisons were performed with univariate analyses (SPSS®) controlling for TIV in volumetric data. Correlations were performed with Spearman correlation test (SPSS®). Voxelwise comparisons were done with non-parametric analysis and statistical significance was set at p < 0.05 (corrected for multiple comparisons).

### Consent for publication

All the authors have approved the manuscript.

## Supplementary information

Supplementary information.

## Data Availability

All data generated and or analyzed during this study are included in this published article and its supplementary information files.
