# Author Correction: Calcium mishandling in absence of primary mitochondrial dysfunction drives cellular pathology in Wolfram Syndrome

**DOI:** 10.1038/s41598-020-67203-2

**Published:** 2020-06-23

**Authors:** Chiara La Morgia, Alessandra Maresca, Giulia Amore, Laura Ludovica Gramegna, Michele Carbonelli, Emanuela Scimonelli, Alberto Danese, Simone Patergnani, Leonardo Caporali, Francesca Tagliavini, Valentina Del Dotto, Mariantonietta Capristo, Federico Sadun, Piero Barboni, Giacomo Savini, Stefania Evangelisti, Claudio Bianchini, Maria Lucia Valentino, Rocco Liguori, Caterina Tonon, Carlotta Giorgi, Paolo Pinton, Raffaele Lodi, Valerio Carelli

**Affiliations:** 1 IRCCS Istituto delle Scienze Neurologiche di Bologna, UOC Clinica Neurologica, Bologna, Italy; 2 Dipartimento di Scienze Biomediche e Neuromotorie, Università di Bologna, Bologna, Italy; 3 IRCCS Istituto delle Scienze Neurologiche di Bologna, UO Diagnostica Funzionale Neuroradiologica, Bologna, Italy; 4 Department of Morphology, Surgery and Experimental Medicine, Section of Pathology, Oncology and Experimental Biology, Laboratory for Technologies of Advanced Therapies (LTTA), University of Ferrara, Ferrara, Italy; 5 Maria Cecilia Hospital, GVM Care & Research, 48033 Cotignola, Ravenna Italy; 6 Ospedale Oftalmico Roma, Rome, Italy; 7 Studio Oculistico D’Azeglio, Bologna, Italy; 8 IRCCS G.B. Bietti Foundation, Rome, Italy

Correction to: *Scientific Reports* 10.1038/s41598-020-61735-3, published online 16 March 2020

In the original version of this Article, Giacomo Savini was incorrectly affiliated with ‘Studio Oculistico d’Azeglio, Bologna, Italy’. The correct affiliation is listed below:

IRCCS G.B. Bietti Foundation, Rome, Italy

The original version of this Article also contained an error in Affiliation 6, which was incorrectly given as ‘Ospedale Oftalmico Roma, Rome, Italy, Rome, Italy’. The correct affiliation is listed below:

Ospedale Oftalmico Roma, Rome, Italy.

Also, the Acknowledgements section in this Article was incomplete.

“We are deeply grateful to the generous collaborations of our WS patients and their families. This study was supported by the Grant GR-2016-02361449 to LC and by the “Ricerca Corrente” funding to VC, both from the Italian Ministry of Health.”

now reads:

“We are deeply grateful to the generous collaborations of our WS patients and their families. This study was supported by the Grant GR-2016-02361449 to LC and by the “Ricerca Corrente” funding to VC, both from the Italian Ministry of Health. The contribution of IRCCS - G.B. Bietti Foundation was supported by Fondazione Roma and the Italian Ministry of Health”

These errors have now been corrected in the PDF and HTML versions of the Article, and in the accompanying Supplementary Information.

