# Supplementary material for: Calcium mishandling in absence of primary mitochondrial dysfunction drives cellular pathology in Wolfram Syndrome
Source: Sci Rep. 2020 Mar 16;10:4785. doi: 10.1038/s41598-020-61735-3 (PMC7075867; doi:10.1038/s41598-020-61735-3)
Supplement: Supplementary file 1 — Supplementary information. [file 41598_2020_61735_MOESM1_ESM.docx]

**Supplementary Information**

**Calcium mishandling in absence of primary mitochondrial dysfunction drives cellular pathology in Wolfram Syndrome**

Chiara La Morgia,^1,2*^Alessandra Maresca,^1*^ Giulia Amore,^2^ Laura Ludovica Gramegna,^2,3^ Michele Carbonelli,^1^ Emanuela Scimonelli,^2^ Alberto Danese,^4^ Simone Patergnani,^4,5^ Leonardo Caporali,^1^ Francesca Tagliavini,^1^ Valentina Del Dotto,^2^ Mariantonietta Capristo,^1^ Federico Sadun,^6^ Piero Barboni,^7^ Giacomo Savini,^8^ Stefania Evangelisti,^2^ Claudio Bianchini,^2^ Maria Lucia Valentino,^1,2^ Rocco Liguori,^1,2^ Caterina Tonon,^2,3^ Carlotta Giorgi,^4^ Paolo Pinton,^4,5#^ Raffaele Lodi,^2,3#^ Valerio Carelli^1,2#^

^1^IRCCS Istituto delle Scienze Neurologiche di Bologna, UOC Clinica Neurologica, Bologna, Italy

^2^Department of Biomedical and Neuromotor Sciences, University of Bologna, Bologna, Italy

^3^IRCCS Istituto delle Scienze Neurologiche di Bologna, UO Diagnostica Funzionale Neuroradiologica, Bologna, Italy

^4^Department of Morphology, Surgery and Experimental Medicine, Section of Pathology, Oncology and Experimental Biology, Laboratory for Technologies of Advanced Therapies (LTTA), University of Ferrara, Ferrara, Italy

^5^Maria Cecilia Hospital, GVM Care & Research, 48033, Cotignola, Ravenna, Italy

^6^ Ospedale Oftalmico Roma, Rome, Italy

^7^Studio Oculistico D’Azeglio, Bologna, Italy

^8^IRCCS G.B. Bietti Foundation, Rome, Italy

*These authors equally contributed to the manuscript

#These authors share senior authorship

**Corresponding author:**

Chiara La Morgia, MD, PhD

IRCCS Istituto delle Scienze Neurologiche di Bologna; Dipartimento di Scienze Biomediche e Neuromotorie; UOC Clinica Neurologica, Ospedale Bellaria

Via Altura, 3 40139 Bologna, Italy

Email: [chiaralamorgia@gmail.com](mailto:chiaralamorgia@gmail.com); [chiara.lamorgia@unibo.it](mailto:chiara.lamorgia@unibo.it)

Tel: +390514966112, Fax: +390514966208

**Supplementary Data Inventory**

**Supplementary Figures**

**Figure S1.** Skeletal muscle biopsies.

**Figure S2.** VBM and TBSS results.

**Supplementary Tables**

**Table S1.** VBM and TBSS results: Wolfram Syndrome patient’s vs HC.

**Table S2.** Regional Subcortical Volumes in Wolfram Syndrome patients and control groups.

**Table S3.** Single Voxel brain ^1^H-MRS metabolite ratios in Wolfram Syndrome patients and matched healthy subjects.

**Original Blots**

**Original Blots Fig 3.** Original blots regarding the data shown in Fig 3A.

**Original Blots Fig 4.** Original blots regarding the data shown in Fig 4H.

**
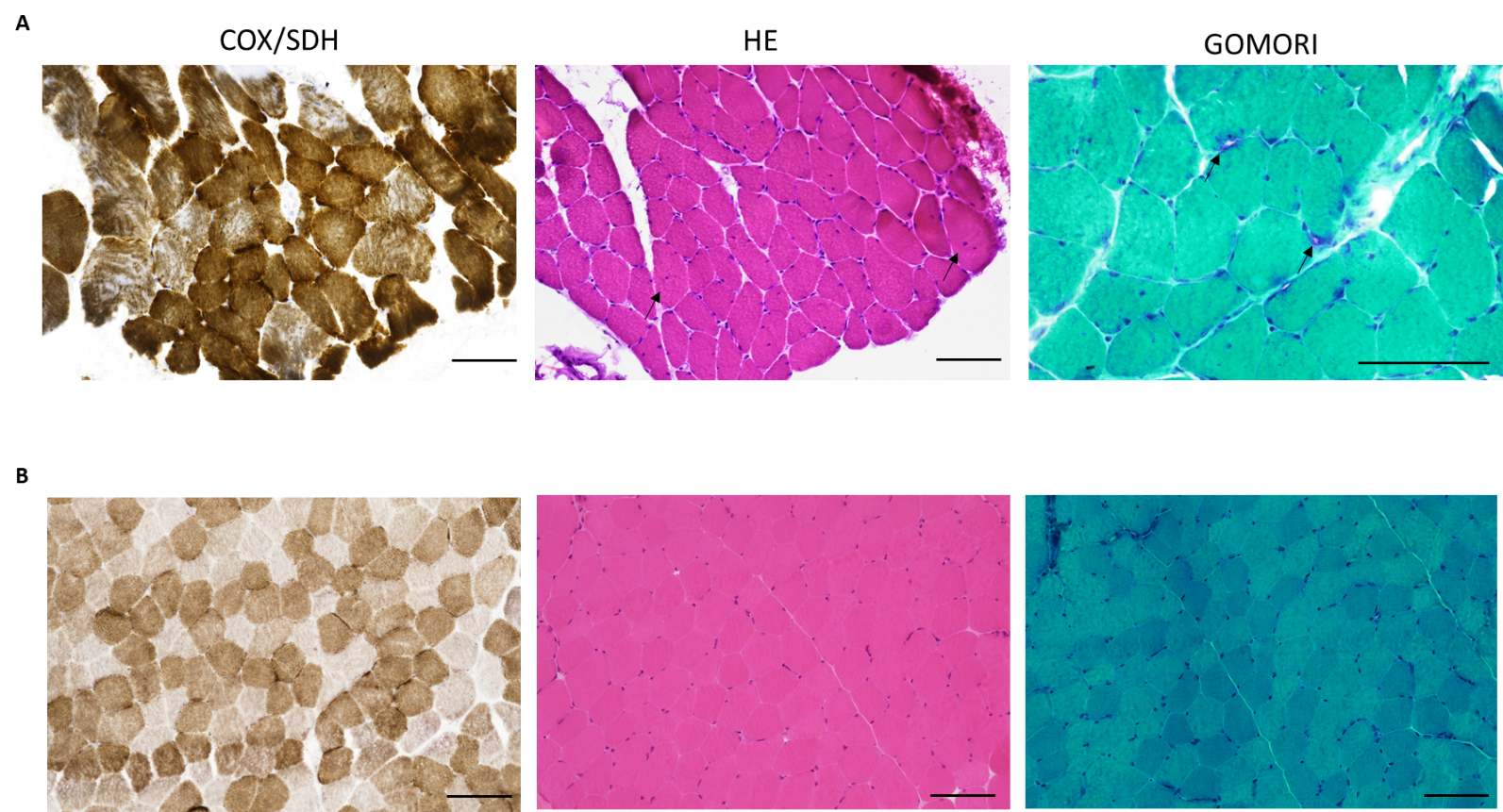
Figure S1.** Skeletal muscle biopsies

COX/SDH, HE and Gomori modified trichrome staining of muscle biopsies from patients n. 4 (A) and n. 12 (B). Arrows indicate fiber with central nuclei in the HE image and rimmed vacuoles in the Gomori image. Bars: 100µm.

**Figure S2.** VBM and TBSS results.
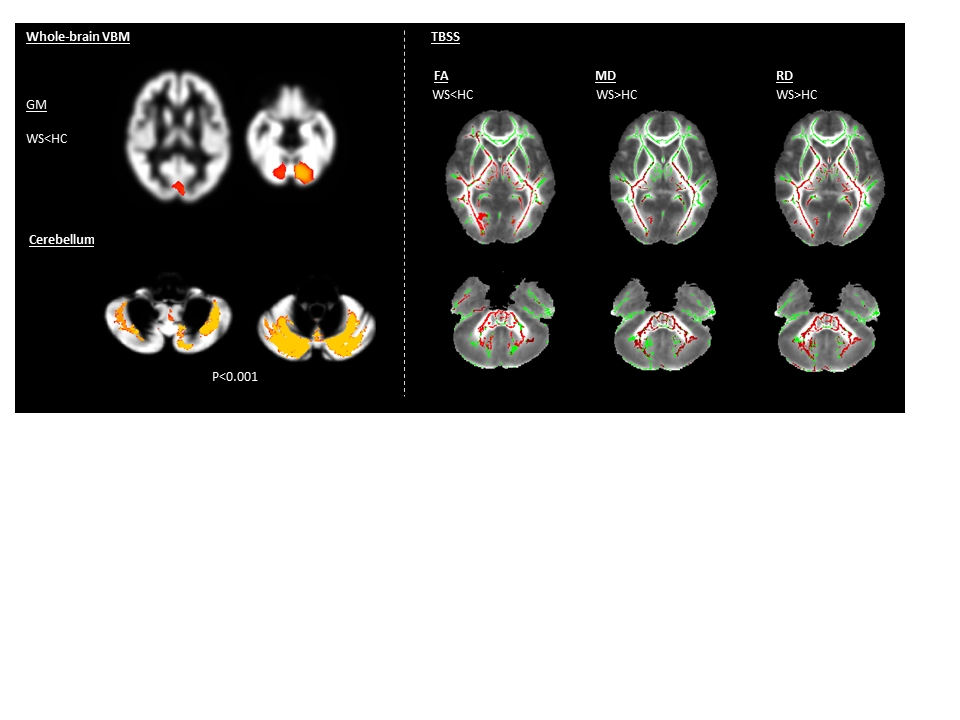


VBM showed cortical grey matter (GM) volume loss in the calcarine cortex (p<0.05, corrected) and in multiple infratentorial structures (p<0.001, corrected) specifically bilateral I-IV, V, VI, VIIb, and left VIIIa; bilateral crus I, II; and vermis VI, VIIIa, IX (Table S1).

TBSS highlighted lower FA (mainly driven by radial diffusivity increase) for patients mostly within optic radiations and optic tracts, posterior limbs of internal capsules; cerebellar peduncles (inferior, middle and superior), and to less extent corpus callosum (p<0.05, corrected, in red; Table S1). Images are shown in radiological convention.

Abbreviations: VBM, Voxel Based Morphometry; TBSS, tract-based spatial statistics; FA, fractional anisotropy; MD, mean diffusivity; RD, radial diffusivity; AD, axial diffusivity; WS, Wolfram syndrome; HC, healthy controls.

| **VBM Cortical Grey Matter** | | **p<0.05** | **Brain regions** | |
| --- | --- | --- | --- | --- |
| WS < HC | |  | Bilateral calcarine cortex | |
|  |  |  | Bilateral cerebellar cortex | |
| WS > HC | | / |  |  |
| **VBM Infratentorial Grey Matter** | | **p<0.001** | **Brain regions** | |
| WS < HC | |  | Bilateral I-IV, V, VI, VIIb, | |
|  |  |  | Left VIIIa  Bilateral crus I, II  Vermis VI, VIIIa, IX | |
|  |  |  |  | |
| WS > HC | | / |  |  |
| **TBSS White Matter** | | **p<0.05** | **Brain regions** | |
| FA | WS < HC |  | Optic radiations | |
|  |  |  | Optic tracts | |
|  |  |  | Posterior limbs of internal capsule | |
|  |  |  | Cerebellar peduncles | |
|  |  |  | Portions of corpus callosum | |
|  | WS > HC | / |  |  |
| MD | WS < HC | / |  |  |
|  | WS > HC |  | Optic radiations | |
|  |  |  | Optic tracts | |
|  |  |  | Posterior limbs of internal capsule | |
|  |  |  | Cerebellar peduncles | |
| AD | WS < HC | / |  |  |
|  | WS > HC | / |  |  |
| RD | WS < HC | / |  |  |
|  | WS > HC |  | Optic radiations | |
|  |  |  | Optic tracts | |
|  |  |  | Posterior limbs of internal capsule | |
|  |  |  | Cerebellar peduncles | |
|  |  |  | Portions of corpus callosum | |
|  |  |  |  |  |
|  |  |  |  |  |

**Table S1**. VBM and TBSS results: Wolfram Syndrome patient’s vs HC.

Cohorts - VBM analysis. WS: N=10 (3M/7F), age (28.8±12.0) y ; HC: N=12 (4M/8F), age (32.4±10.8) y. Cohorts - TBSS analysis. WS: N=9 (2M/7F), age (26.4±9.94) y; HC: N=12 (4M/8F), age (32.4±10.8) y. Legend: WS, Wolfram syndrome; HC, healthy controls; VBM, Voxel Based Morphometry; TBSS, tract-based spatial statistics; FA, fractional anisotropy; MD, mean diffusivity; RD, radial diffusivity; AD, axial diffusivity; /, not significant. Results are corrected for multiple comparisons.

| **Subcortical Regions Volumes** | **p-value** | **WS (n=10, 29 ± 12 yrs, 3M)**  **group mean ± SD** | **HC (n=12, 32 ± 11 yrs, 4M)**  **group mean ± SD** |
| --- | --- | --- | --- |
| Left Lateral Ventricle | 0,361 | 6655 (4899) | 5959 (2477) |
| Left Inferior Lateral Ventricle | 0,151 | 425 (366) | 283 (147) |
| Left Cerebellum White Matter | **<0.0001** | 8351 (1397) | 13238 (1519) |
| Left Cerebellum Cortex | **0,003** | 38768 (5583) | 48326 (3590) |
| Left Thalamus | **0,003** | 5471 (712) | 7316 (1000) |
| Left Caudate | 0,503 | 3096 (491) | 3429 (593) |
| Left Putamen | 0,800 | 4667 (1087) | 5500 (848) |
| Left Pallidum | 0,412 | 1270 (258) | 1519 (216) |
| 3rd Ventricle | 0,254 | 1048 (469) | 907 (222) |
| 4th Ventricle | **0,015** | 2287 (814) | 1695 (286) |
| Brainstem | **<0.0001** | 13382 (2150) | 20752 (2130) |
| Left Hippocampus | 0,294 | 3360 (349) | 3805 (471) |
| Left Amygdala | 0,984 | 1197 (247) | 1390 (250) |
| Left Accumbens | 0,624 | 561 (161) | 598 (116) |
| Left Ventral DC | **<0.0001** | 2586 (270) | 3660 (532) |
| Right Lateral Ventricle | 0,277 | 6400 (4627) | 5838 (2507) |
| Right Inferior Lateral Ventricle | 0,266 | 424 (319) | 370 (268) |
| Right Cerebellum White Matter | **<0.0001** | 8877 (1708) | 13680 (1459) |
| Right Cerebellum Cortex | **0,001** | 39751 (4647) | 49315 (3512) |
| Right Thalamus | **0,002** | 5044 (924) | 6994 (856) |
| Right Caudate | 0,553 | 3284 (448) | 3570 (658) |
| Right Putamen | 0,792 | 4736 (833) | 5430 (706) |
| Right Pallidum | 0,610 | 1308 (243) | 1557 (273) |
| Right Hippocampus | 0,077 | 3443 (413) | 3896 (268) |
| Right Amygdala | 0,818 | 1305 (263) | 1505 (319) |
| Right Accumbens | 0,389 | 526 (141) | 623 (99) |
| Right Ventral DC | **<0.0001** | 2669 (274) | 3772 (458) |
| Corpus Callosum | 0,341 | 2487 (416) | 3116 (459) |

**Table S2**. Regional Subcortical Volumes in Wolfram Syndrome patients and control groups.

Cohorts. WS: N=10 (3M/7F), age (28.8±12.0) y ; HC: N=12 (4M/8F), age (32.4±10.8) y. Legend: Freesurfer significant (p<0.05) results are reported in bold; comparisons that survived Bonferroni correction for multiple comparisons (p < 0.0017) are also underlined.

**Table S3**. Single Voxel brain ^1^H-MRS metabolite ratios in Wolfram Syndrome patients and matched healthy subjects.

| **^1^H-MRS Localisation** | | **p-value** | **WS** | **Healthy controls** | |
| --- | --- | --- | --- | --- | --- |
| **Cerebellar hemisphere** | |  | **n=9, (29 ± 12) y, 3M** | **n=10, (30 ±10) y, 4M** | |
|  | NAA/Cr | **0.003** | 0.97 (0.23) | 1.32 (0.21) | |
|  | mI/Cr | **0.012** | 0.83 (0.12) | 0.67 (0.14) | |
|  | NAA/mI | **<0.0001** | 1.19 (0.33) | 2.07 (0.52) | |
| **Pons** | |  | **n=5, (31± 11) y, 0M** | **n=5, (30 ± 11) y, 0M** | |
|  | NAA/Cr | **0.004** | 1.80 (0.34) | 2.74 (0.42) | |
|  | mI/Cr | 0.211 | 1.72 (0.31) | 1.49 (0.21) | |
|  | NAA/mI | **0.005** | 1.09 (0.36) | 1.85 (0.26) | |
| **POWM** | |  | **n=7, (31 ± 14) y, 2M** | **n=7, (31 ± 12) y, 2M** | |
|  | NAA/Cr | 0.752 | 1.89 (0.20) | 1.92 (0.14) | |
|  | mI/Cr | 0.092 | 1.00 (0.20) | 0.82 (0.17) | |
|  | NAA/mI | 0.083 | 1.96 (0.41) | 2.42 (0.51) | |
|  |  |  |  | |  |

Legend: data is reported as (mean ± SD) years; ^1^H-MRS significant (p<0.05) results are in bold; comparisons that survived Bonferroni correction for multiple comparisons (p<0.017) are also underlined.

y= years; M= male; POWM= parieto-occipital white matter


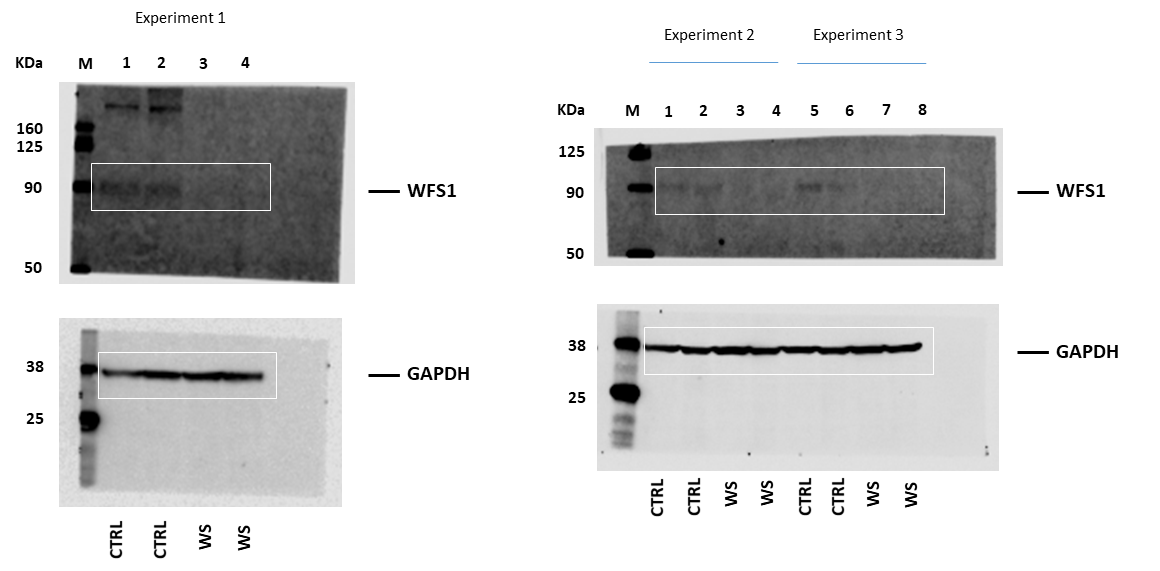
**Original Blots Fig 3.** Original blots regarding the data shown in Fig 3A.

✄

✄

The original blots relative to Fig. 3A in the main manuscript are shown here. Three independent samples for each cell line were analyzed; replicates 2 and 3 were run in the same gel. The membranes were cut at 50kDa after blocking; the upper parts were incubated with primary antibody for WFS1 and the lower parts were incubated with primary antibody for GAPDH. Secondary antibodies used are IRDye 800 CW Goat anti mouse (FE30926210) and IRDye 680RD Goat anti Rabbit (FE3680710). Images were acquired using the LI-COR Odyssey Fc Dual Mode imager.

**Original Blots Fig 4.** Original blots regarding the data shown in Fig 4H.


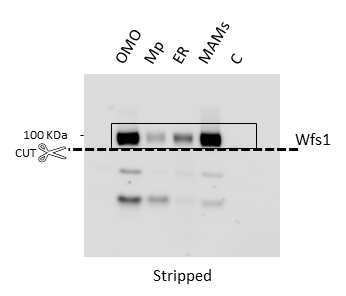

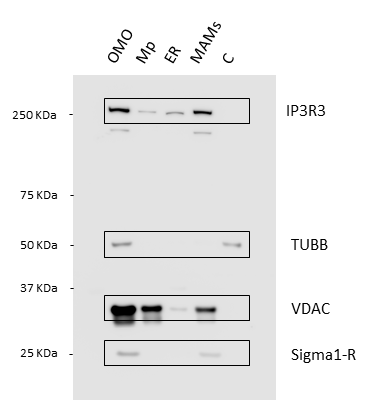


**C**

**A**

**B**


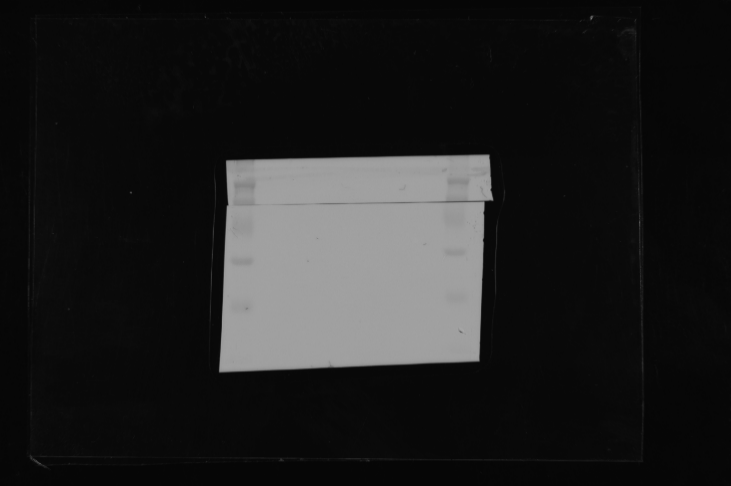


The original blots relative to Fig. 4H in the main manuscript are shown here. The membrane was first blotted with primary antibodies for IP3R3, TUBB, VDAC, Sigma1-R **(A)**. The membrane was then stripped, cut and blotted with primary antibodies for Wfs1 and for another protein not shown in this article **(B-C)**. Secondary antibodies used were Goat anti- Mouse IgG (H+L) Secondary Antibody, HRP (Thermo Scientific, catalogue num- ber 32430), Goat anti-Rabbit IgG (H+L) Secondary Antibody, HRP (Thermo Scientific, catalogue number 32460). The immunoreactive bands were acquired using the ImageQuant LAS-4000 System (GE Healthcare).
